# The impact of zeaxanthin and lycopene on fatty acid beta-oxidation in isolated peroxisomes from frozen livers of obese rats

**DOI:** 10.3389/fmolb.2026.1906904

**Published:** 2026-09-23

**Authors:** Lina Baz, Mona Al-Thepyani

**Affiliations:** 1 Department of Biochemistry, Faculty of Science, King Abdulaziz University, Jeddah, Saudi Arabia; 2 Department of Chemistry, College of Sciences & Arts, King Abdulaziz University, Rabigh, Saudi Arabia; 3 Neuroscience and Geroscience Research Unit, King Fahd Medical Research Center, King Abdulaziz University, Jeddah, Saudi Arabia

**Keywords:** peroxisomes, peroxisomes isolation, high-fat diet, peroxisomal β-oxidation, fatty acid oxidation system (FAOS), zeaxanthin, lycopene, GC/MS fatty acid analysis

## Abstract

Over the past decade, research has emphasized the role of organelle dysfunction in various diseases, particularly obesity and related metabolic disorders. Peroxisomes, essential for lipid metabolism and redox balance, have been associated with obesity-related complications. Although carotenoids such as zeaxanthin (Zi) and lycopene (Ly) are known for their antioxidant and anti-inflammatory properties, their effects on peroxisomal function in obesity remain unexamined. This study evaluated the effects of Zi and Ly supplementation on peroxisomal β-oxidation, oxidative stress, and inflammatory markers in rats fed a high-fat diet (HFD). Forty male Wistar rats were divided into four groups: normal diet, HFD, Zi (100 mg/kg), and Ly (25 mg/kg). After a 12-week HFD regimen, the rats received Zi and Ly for an additional 5 weeks. Peroxisome function was assessed by measuring the activity of marker enzymes such as catalase and acyl-CoA oxidase, along with evaluations of antioxidant enzyme activity, oxidative stress indicators, inflammatory biomarkers, and lipid profiles. Serum fatty acid content was analyzed using gas chromatography/mass spectrometry (GC/MS). Results showed that HFD intake resulted in significant weight gain and peroxisomal dysfunction, indicated by reduced enzyme activities and increased serum saturated fatty acids. In contrast, Zi and Ly treatments improved peroxisomal β-oxidation, altered fatty acid profiles, decreased oxidative stress and inflammation markers, and enhanced antioxidant enzyme activities. These findings suggest that Zi and Ly can restore peroxisomal function impaired by high-fat diets, highlighting peroxisomal homeostasis as a potential therapeutic target in obesity. Further research is recommended to elucidate the underlying molecular mechanisms.

## 1 Introduction

Obesity is a complex disease that has a multifactorial etiology, including complex interactions between environmental factors, human behavior and genetic susceptibility. It is responsible for increasing the risk of developing diseases such as dyslipidemia and several metabolic disorders (Blüher, 2019), (De Lorenzo et al., 2019).

Diet can affect a variety of aspects of human health and a high-fat diet (HFD) is associated with an epidemic of obesity and related chronic metabolic diseases (Bertoncini‐Silva et al., 2023), (Li et al., 2016), (Sun et al., 2022). Consuming HFD leads to fat accumulation associated with systemic oxidative stress in humans and rodents through increased reactive oxygen species (ROS) production and oxidative stress. This increase in oxidative stress leads to lipid peroxidation, causing an imbalance in the production of adipocytokines and other inflammatory factors (Kesh et al., 2016), (Syarif et al., 2024) (Figure 1).

**FIGURE 1:**
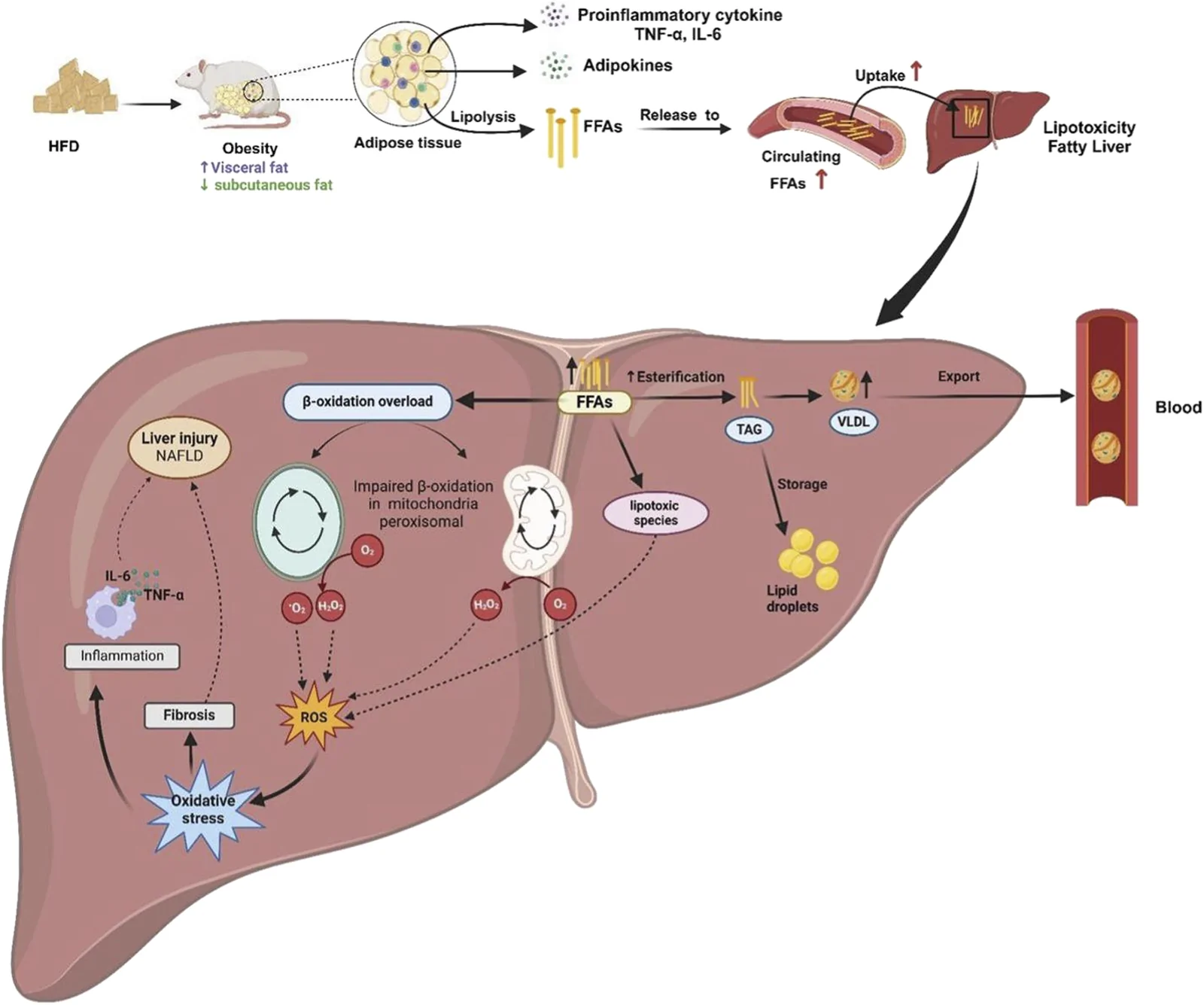
The proposed mechanism of HFD for fat accumulation and lipid metabolism disorders. HFD: high fat diet; TNF-α: tumor necrosis factor; IL-6: interleukin 6; FFAs:free fatty acids; TGA:Triacylglycerol; VLDL:Very low density lipoprotein; ROS: reactive oxygen species; NAFLD: Non-alcoholic fatty liver disease.

Cellular organelles not only form the basic structure of the cell but are also important for maintaining normal physiological activities. Their dysfunction or impaired interactions often lead to various diseases (Hu et al., 2023). Peroxisomes are cellular organelles present in virtually all eukaryotic cells and play an important role in numerous processes related to lipid metabolism (Fransen et al., 2017), (Fujiki and Bassik, 2021), (Just et al., 2014) (Wanders et al., 2023). Peroxisomes play a role in various metabolic pathways including the α-oxidation of fatty acids, β-oxidation of very-long-chain fatty acids, glyoxylate detoxification and polyamine and purine degradation. Additionally, peroxisomes are involved in cellular redox metabolism by generating and degrading hydrogen peroxide (H_2_O_2_)in the matrix (Lizard, 2020). Peroxisomes are thought to be responsible for up to 35% of total (H_2_O_2_) production in mammalian tissues (Boveris et al., 1972), (Liu et al., 2019a). Catalase is a marker of peroxisome function and plays a critical role in protecting peroxisomes from peroxide formation and toxicity (Antonenkov et al., 2010), (Siraki et al., 2002), (Walton et al., 2017). Moreover, acyl-CoA oxidase is another peroxisome enzyme that catalyzes the first and rate-determining step of peroxisomal β-oxidation of fatty acids and is a major producer of hydrogen peroxide (Ferdinandusse et al., 2007a), (He et al., 2020a).

Obesity impacts cellular organelle function (Borchers et al., 2025), (Kleiboeker and Lodhi, 2022a). Recent studies reveal that obesity severely impairs the structural and functional integrity of adipocyte peroxisomes, likely intensifying inflammation in white adipose tissue and systemic metabolic dysregulation (Choi et al., 2025). The decline in peroxisomal function impairs the breakdown of pro-inflammatory lipid mediators, including leukotrienes, and thus exacerbates oxidative stress (Delille et al., 2006). Inhibition of peroxisomal biogenesis creates a harmful feedback loop, where increased hydrogen peroxide and TNF-α concentrations further reduce peroxisomal function (Jiang, 2019), (Piao et al., 2019a). Liver peroxisomal dysfunction parallels these clinical changes, as chronic lipid overload in hepatocytes impairs peroxisomal β-oxidation and facilitates the progression of non-alcoholic fatty liver disease (Jiang et al., 2018). Furthermore, along with these metabolic changes, the declining effectiveness of peroxisomal signaling mechanisms further weakens cellular stress responses, accelerating the development of many age-related and metabolic diseases (Cipolla and Lodhi, 2017), (Kumar et al., 2024a).

Isolating peroxisomes is crucial for characterizing their constituents and functions (Wanders et al., 2023). Rat liver supplied the fractions utilized by De Duve and colleagues for the early biochemical characterisation of peroxisomes (Baudhuin et al., 1965), (De Duve and Baudhuin, 1966). In 1968, Leighton and colleagues developed techniques to isolate peroxisomes from rat liver (Leighton et al., 1968). Advances in science and technology have since improved the methodology for isolating peroxisomes. To date, all techniques for isolating peroxisomes have been based on fresh tissue samples. The only experimental study to separate peroxisomes from frozen human liver specimens was conducted in 1992 by (Alvarez et al., 1992). This methodological approach is particularly important for clinical obesity research, as it enables the study of peroxisomal characteristics in stored biopsy samples when fresh tissue is unavailable. Beyond this basic research, there is limited information on the isolation of organelles from frozen samples. Recent studies have aimed to expand on these findings by using differential and iodixanol gradient centrifugation to isolate peroxisomes specifically from frozen rat liver tissue (Baz et al., 2023).

Fatty acids are a complex family of bioactive molecules their metabolism may occur in several cell compartments, but β-oxidation of these lipids is restricted only in mitochondria and peroxisomes (Fransen et al., 2017) (Wanders et al., 2023), (Lizard, 2020), (Demarquoy and Borgne, 2014). Short chain fatty acids, medium chain fatty acids and long chain fatty acids are oxidized mainly in mitochondria and to a lesser extent in peroxisomes (Violante et al., 2019). While very long chain fatty acids (VLCFA) are primarily exclusively metabolized in peroxisomes by β-oxidation, as shown by elevated plasma VLCFA levels in various diseases of peroxisome biogenesis (Wanders, 2014), (Waterham et al., 2016). Moreover, peroxisomes can process long chain fatty acids and reduce metabolic stress when mitochondria are overloaded (Austin and St-Pierre, 2012), (Noland et al., 2007). VLCFAs are generally very toxic when free *in vivo*, and their oxidative metabolism depends on peroxisomal β-oxidation. Most studies have focused on understanding the mechanism of β-oxidation in mitochondria, while the importance of peroxisomal β-oxidation has been overlooked for a long time (Liu et al., 2019b), (Lu et al., 2022a).

Currently, there are many therapeutic options for the treatment of obesity, including dietary changes, physical activity, behavioral modification, surgical intervention and drugs (Williams et al., 2020). Due to the undesirable side effects of anti-obesity medications and the numerous risks associated with surgical interventions, the combination of regular exercise and a healthy diet remains the safest and most cost-effective method for obesity management (Ammar et al., 2017), (Bradley et al., 2008). Furthermore, since natural products such as polyphenols, capsaicinoids, curcuminoids and carotenoids are safe and non-toxic, consuming functional food is one of the best (Mounien et al., 2019), (Paetau et al., 1998).

Carotenoids are natural pigments found in fruits, vegetables, algae, and photosynthetic bacteria. Carotenoids can be divided into two groups according to their chemical structure: carotenes, which are hydrocarbons such as lycopene and xanthophylls like zeaxanthin, which contain oxygen and are therefore more polar (Mounien et al., 2019), (Paetau et al., 1998), (Eggersdorfer and Wyss, 2018). Carotenoids play a central role in the biology of adipose tissue, can inhibit the accumulation of body fat and prevent or treat obesity (Bonet et al., 2015).

Zeaxanthin (Zi) and lycopene (Ly) are types of carotenoid pigment which present in a wide range of fruits and vegetables (Antonenkov et al., 2010), (Bouyahya et al., 2021), (Murillo et al., 2019). Zeaxanthin is found in foods such as corn, cantaloupe, orange, carrots, or yellow peppers, salmon,fish and eggs (Eisenhauer et al., 2017), while lycopene is found in tomatoes, pink grapefruit, watermelon and papaya (Khan et al., 2021a). Research has demonstrated that when administered orally, Zi improved dyslipidemia and slowed the progression of obesity in mice (Liu et al., 2017). Tuzcu and coworkers found that rats fed HFD showed increased oxidative stress and upregulation of inflammatory markers. However, the administration of Zi significantly reduced oxidative damage by increasing the activity of antioxidant enzymes such as superoxide dismutases (SOD) and catalase (CAT) and reducing malondialdehyde (MDA) concentration (Tuzcu et al., 2017). On the other hand, Ly has attracted the attention of nutritionists, clinicians and research scientists for its potential applications in the management of various human diseases, due to its antioxidant, anti-inflammatory effect, anticancer and cardiovascular protective effects (Khan et al., 2021b). Ly may preserve redox homeostasis and suppress the overproduction of pro-inflammatory factors (Ip et al., 2015). Ly reduces the levels of tumor necrosis factor (TNF-α) and MDA, improves the status of enzymatic (SOD, CAT and peroxidase) in the cell and acts as an important antioxidant (Khan et al., 2021b), (Ip et al., 2015), (Róvero Costa et al., 2019). In addition, Ly treatment reduced ROS generation (Chen et al., 2019). The beneficial effect of tomato powder supplementation was also related to the pro-inflammatory markers such as cytokines interleukin 6 (IL-6) and TNF-α, adipokines (resistin and leptin) (Fenni et al., 2017). In brief, Ly restores impaired liver function through the promotion of fatty acid β-oxidation and inhibition of lipogenesis via attenuating inflammatory and ROS stimulation. The current study aims to explore the potential effects of Zi and Ly on peroxisomal β-oxidation by evaluating lipid profiles, inflammatory markers, oxidative stress biomarkers, and antioxidant enzyme activities in high-fat diet-induced obese rats.

## 2 Materials and methods

### 2.1 Materials

#### 2.1.1 Chemicals

Zeaxanthin (Zi) and lycopene (Ly) (purity of 98%) were provided by Xi’an Tongze Biotech Co, Ltd, Xi’an, China. Zi was extracted from marigold flowers, while Ly was extracted from tomatoes. OptiPrep 60% (w/v) iodixanol density gradient medium was purchased from Sigma-Aldrich (St. Louis, MO, United States of America). 3-(N-morpholino) propanesulfonic acid (MOPS) and ethylenediaminetetraacetic acid (EDTA) were purchased from Beijing Solarbio Science and Technology Co., Ltd. In Beijing, China. Sucrose, sodium hydroxide (NaOH) and ethanol were obtained from commercial sources. All chemicals used were of analytical grade.

#### 2.1.2 Equipment

Potter–Elvehjem tissue homogenizer with a loose-fitting pestle (distance 0.1–0.15 mm, volume 30 mL), polyallomer quick-seal tubes (39 mL, Beckman Coulter, Brea, CA, United States of America), ultracentrifuge and fixed-angle rotor (Himac-CP100WX, P80AT-0135 rotor, Tokyo, Japan), spectrophotometer (Thermo Scientific GENESYS 10S Visible and UV/Visible, United States of America), vortex (Thermo Scientific, China), refrigerated low and high-speed centrifuge (HERMLE Labortechnik GmbH, Gosheim, Germany were used.

#### 2.1.3 Animals

In this study, forty male Wistar rats aged 7 weeks, with an average body weight of 200 ± 10 g, were obtained from the Animal House Department of King Fahad Medical Research Center (KFMRC), Jeddah, Saudi Arabia. The animals were housed in polycarbonate cages (4 groups) with 10 rats each in a room with 12/12 h of light/darkness, a humidity of 50% and a temperature of 22 °C ± 2 °C. A balanced commercial diet and tap water were provided throughout the experiment. The study was approved by the Ethics Committee of King Abdulaziz University (Approval No. 518-20) and conducted according to the rules and regulations of the Animal Care and Use Committee (KFMRC).

### 2.2 Methods

#### 2.2.1 Preparation of high-fat diet (HFD)

A standard chow diet (Grain Silos & Flour Mills Organization, KSA) was obtained from the KFMRC. The standard diet was composed of carbohydrates (67.8%), protein (20%) and fat (3.5%). The high-fat diet (HFD) was prepared according to the protocol of (Gheibi et al., 2017). The HFD was composed of carbohydrates (40.4%), protein (19.4%) and fat (32.2%). All the ingredients were weighed (as mentioned in (Table 1) and mixed thoroughly. Melted lard was added to obtain the right consistency and finally, the mixture was shaped into pellets and was stored at 4 °C for 1 week.

**TABLE 1:** Ingredients used for the preparation of high-fat diet (HFD).

Ingredients	Diet (g/kg)
Powdered NPD	596
Commercial butter	298
Casein	74
DL-methionine	2
Vitamin and mineral mix	30

NPD:normal pellets diet.

#### 2.2.2 Preparation of Zi and Ly dose selection

Zi and Ly were mixed with sunflower oil and stored in the dark at 4 °C until use (Pierine et al., 2014a), (Sindhu et al., 2010). The prepared Zi and Ly sunflower oil mixtures were stirred for 20 min and warmed in a water bath at 37 C before feeding to the animals. The dose of 100 mg/kg Zi was chosen based on numerous *in vivo* studies (Tuzcu et al., 2017), (Sindhu and Kuttan, 2012), (Xue et al., 2015). While the dose of 25 mg/kg Ly was selected according to (Pierine et al., 2014a), (Yang et al., 2018), (Zuorro et al., 2018).

#### 2.2.3 Experimental design and animal treatment

After 1 week of acclimatization, 40 rats were randomly divided into two separate groups and received either a normal diet (ND; n = 10) or a high-fat diet (HFD; n = 30). After the 12th week, once the difference in body weight difference, the obese animals were randomly divided into a control group and two treated groups (Figure 2). As a result, the following 4 groups (n = 10/group) were kept alive for 5 weeks:

Group I: ND -fed a normal pellet diet
Group II: HFD (fed normal pellet diet only)
Group III: HFD + Zi (Oral administration of zeaxanthin, 100 mg/kg/day).
Group IV: HFD + Ly (Oral administration of lycopene, 25 mg/kg/day).

**FIGURE 2:**
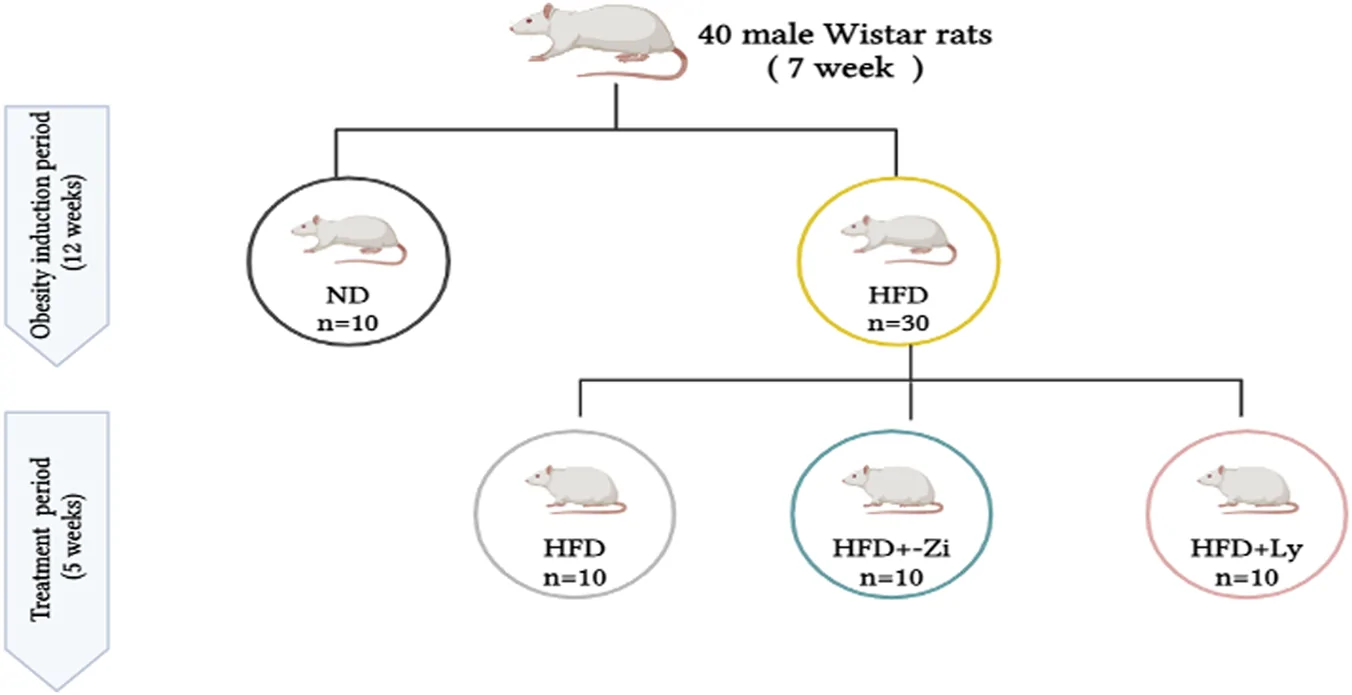
Study design. The rats were randomized into two groups: Normal Dite group (n = 10) and high-fat diet (HFD; n = 30) for 12 weeks (obesity induction). Subsequently, the HFD group was divided into three groups (n = 10/group): HFD, HFD + Zi and HFD + Ly (intervention period).

All obese groups were continually fed the HFD during the experimental period (17 weeks) and all groups received the same volume of sunflower oil (∼2 mL/kg/day).

#### 2.2.4 Sampling and tissue preparation

At the end of the experiment, rats fasted overnight and were anesthetized with isoflurane (initiated with 5% isoflurane and maintained at 3% in an oxygen flow of 2 L/min in a sealed container). Blood was drained from the retro-orbital veins of the rats. Serum was collected by centrifugation at 3000 rpm for 15 min and stored at −80 °C for biochemical analysis. The rats were sacrificed by an overdose of isoflurane. The abdominal cavity was then opened, and the liver and adipose tissue were removed, rinsed with 0.9% NaCl, weighed, frozen and stored at −80 °C until analysis.

#### 2.2.5 Body weight and abdominal fat

At the beginning of the experiments, body weight was recorded and then monitored weekly. At the end of the experiments, the weight of abdominal fat was also measured.

#### 2.2.6 Redox homeostasis biomarkers

Markers of oxidative stress, malondialdehyde (MDA), and the activities of the antioxidant enzymes catalase (CAT) and superoxide dismutase (SOD) (Cat. No. BC0025, Cat. No. BC0205, and Cat. No. BC0175, respectively) were determined in hepatic tissue using colorimetric assay kits from Solarbio Science & Technology (Beijing, China) following the manufacturer’s instructions. Briefly, To determine MDA, liver tissue homogenates were subjected to the thiobarbituric acid reaction, producing a chromogenic MDA–TBA complex. Absorbance was measured at 532 nm, and MDA concentrations were obtained from a standard curve and expressed as nmol/g of tissue. Catalase (CAT) activity was evaluated by measuring the rate of hydrogen peroxide (H_2_O_2_) breakdown. The decline in H_2_O_2_ concentration was measured spectrophotometrically at 240 nm. CAT activity was determined in accordance with the manufacturer’s instructions and expressed as U/g tissue. SOD activity was evaluated using an inhibition-based colorimetric method. The chromogenic substrate is degraded by superoxide radicals formed in the reaction system, and the effect of SOD in blocking this reaction was assessed by measuring absorbance at 560 nm. SOD activity was determined in accordance with the manufacturer’s specifications and expressed as U/g tissue.

#### 2.2.7 Proinflammatory cytokines

The concentrations of interleukin-6 (IL-6) and tumor necrosis factor-α (TNF-α) (Cat. No. SEKR-0009 and SEKR-0005, respectively) in serum were determined using rat enzyme-linked immunosorbent assay (ELISA) kits (Solarbio Science & Technology, Beijing, China) according to the manufacturer’s instructions. An aliquot of 100 µL of serum samples and standards was added to the wells of a pre-coated microplate and incubated at 37 °C for 90 min. After aspiration and washing, 100 µL of biotin-conjugated anti-rat IL-6 or TNF-α antibody working solution was added to each well and incubated at 37 °C for 60 min. After washing, 100 µL of streptavidin-tagged detection reagent was added, and the plate was incubated at 37 °C for 30 min. The wells were rinsed three times, after which 100 µL of substrate solution was added. After a 15-min incubation, 50 µL of stop solution was added to terminate the reaction. The optical density of each well was immediately recorded at 450 nm with a microplate reader (BioTek Instruments, Inc., Winooski, VT, United States of America). Cytokine concentrations were calculated from sample absorbance values relative to the standard calibration curve and expressed as pg/mL serum.

#### 2.2.8 Serum lipid profile

Triacylglycerol (TGA) and high-density lipoprotein (HDL) levels in serum were measured using commercial colorimetric assay kits (Cat. No. BC0625 and Cat. No. BC5325, respectively) from Solarbio Science & Technology, Beijing, China. For TAG assessment, serum specimens were mixed with the supplied working reagents, and triglycerides were enzymatically hydrolyzed to yield glycerol. The resulting substances reacted with chromogenic agents to produce a colored molecule. Absorbance was measured at 420 nm with a microplate reader, and TAG concentrations were determined from the standard curve and reported in mmol/L. To determine HDL-C levels, serum specimens were treated with assay reagents that specifically precipitated non-HDL lipoproteins, thereby enabling quantification of cholesterol in HDL particles. Cholesterol in HDL was enzymatically converted via a colorimetric reaction, and the resulting color intensity was measured at 546 nm using a microplate reader. HDL-C levels were determined from the appropriate standard curve and reported in mmol/L. Very low density lipoprotein (VLDL) values were calculated based on the Friedewald equation (Friedewald et al., 1972).

VLDL=TGA/5

#### 2.2.9 Hepatic free fatty acid

Free fatty acids (FFAs) content in hepatic tissue was measured using colorimetric assay kits (Cat. No. BC0595) from Solarbio Science & Technology (Beijing, China), following the manufacturer’s instructions. Briefly, approximately 0.1 g of hepatic tissue was homogenized in 1.0 mL of extraction buffer and centrifuged at 8,000 × g for 10 min at 4 °C. The resulting supernatant was used for analysis. The assay is based on the formation of copper–fatty acid complexes following the binding of FFAs to Cu^2+^ ions. Residual copper ions were measured colorimetrically, with absorbance recorded at 550 nm using a 96-well microplate reader. FFA levels were calculated using a palmitic acid standard curve, in accordance with the manufacturer’s instructions, and expressed as µmol/g of tissue.

#### 2.2.10 Isolation of peroxisome from frozen liver

In this research, we separated the peroxisomes using the protocol we developed with Baz and colleagues, which was published in a journal specializing in separation techniques (Baz et al., 2023). In this study, the purity and efficiency of the isolated peroxisomal fraction were validated using organelle-specific marker enzyme assays, which showed negligible cross-contamination with other subcellular organelles. Therefore, the previously established isolation process was employed in this work to ensure the reproducibility and integrity of the peroxisomal preparations. In our protocol, the frozen liver was used as the starting material based on the study by (Alvarez et al., 1992) who isolated the peroxisome from human frozen liver. Unlike other protocols that use fresh liver samples (Graham, 2002a), (Graham, 2002b), (Islinger et al., 2012), (Manner et al., 2017). This is the first protocol in which peroxisomes have been isolated from frozen rat liver. Therefore, this protocol has been optimized, and the integrity of the isolation and purification process has been evaluated, confirmed, and published (Baz et al., 2023). The protocol used in this study to isolate peroxisomes is based on the method of Graham and Islinger with some modifications (Graham, 2002a), (Graham, 2002b), (Islinger et al., 2012). The isolation of peroxisomes is carried out in three steps: (1) homogenization of the liver tissue with a Potter-Elvenheijm homogenizer at low speeds. As shown in Figure 3, peroxisomes were isolated from frozen liver samples using a three-step protocol comprising tissue homogenization, differential centrifugation, and iodixanol density gradient ultracentrifugation. Liver samples (10–12 g) were washed in ice-cold homogenization buffer (HB; 0.25 M sucrose, 1 mM EDTA, 5 mM MOPS, 0.1% ethanol, pH 7.2), minced into ∼2.5 mm^3^ fragments, and resuspended in HB (3 mL/g tissue). All procedures were performed at 4 °C, and tissue was homogenized using a Potter-Elvehjem homogenizer fitted with a loose-fitting pestle (10 strokes). Subcellular fractionation was then performed by differential centrifugation according to a classical protocol, yielding nuclear, heavy mitochondrial (HM), light mitochondrial (LM), and light mitochondrial-peroxisomal (LM-P) fractions. The homogenate was first centrifuged at 750 × g for 10 min at 4 °C to remove nuclei and cellular debris; the pellet was re-extracted and re-centrifuged to maximize yield. The combined supernatants were then centrifuged at 3500 × g for 10 min at 4 °C to sediment heavy mitochondria; the pellet was subjected to a repeat extraction, and the pooled postmitochondrial supernatants were centrifuged at 23,000 × g for 20 min at 4 °C to obtain the LM-P fraction, which was washed by resuspension and re-centrifugation before final resuspension in HB (3 mL/g pellet) for density gradient ultracentrifugation. For peroxisome purification, 8 mL of the LM-P fraction was layered onto iodixanol gradients, the tubes were filled to capacity with HB, sealed, and ultracentrifuged at 105,000 × g for 1 h 30 min at 4 °C. Three distinct bands formed near the bottom of the tube, with the peroxisomal fraction sedimenting closest to the base, and individual fractions were collected by needle aspiration. After the isolation process, the isolated peroxisome fraction was used to evaluate the efficiency of peroxisomal function by determining the activity of catalase (Baudhuin, 1974), acyl Co-A oxidase (Reubsaet et al., 1988), (Small et al., 1985) and fatty acid oxidation system (FAOS) (Lazarow, 1981) (Figure 3). Peroxisomal catalase activity was measured using a colorimetric method based on the decomposition of hydrogen peroxide. Briefly, tissue specimens were incubated with a reaction mixture containing hydrogen peroxide, and the reaction was stopped by adding a titanium sulfate solution, which reacts with the remaining hydrogen peroxide to form a yellow complex. Absorbance was measured at 405 nm with a spectrophotometer, and catalase activity was expressed in kU/L. Acyl-CoA oxidase (ACOX) activity was determined using a spectrophotometric assay based on the generation of hydrogen peroxide during the oxidation of palmitoyl-CoA. Hydrogen peroxide was detected using leuco-dichlorofluorescein and horseradish peroxidase, and the increase in absorbance was recorded at 502 nm. Enzyme activity was expressed in U/L. The functionality of the peroxisomal fatty acid β-oxidation system (FAOS) was assessed by quantifying the conversion of NAD to NADH during the β-oxidation of palmitoyl-CoA. NADH formation was monitored spectrophotometrically at 340 nm, and FAOS activity was expressed in U/L following correction for background activity.

**FIGURE 3:**
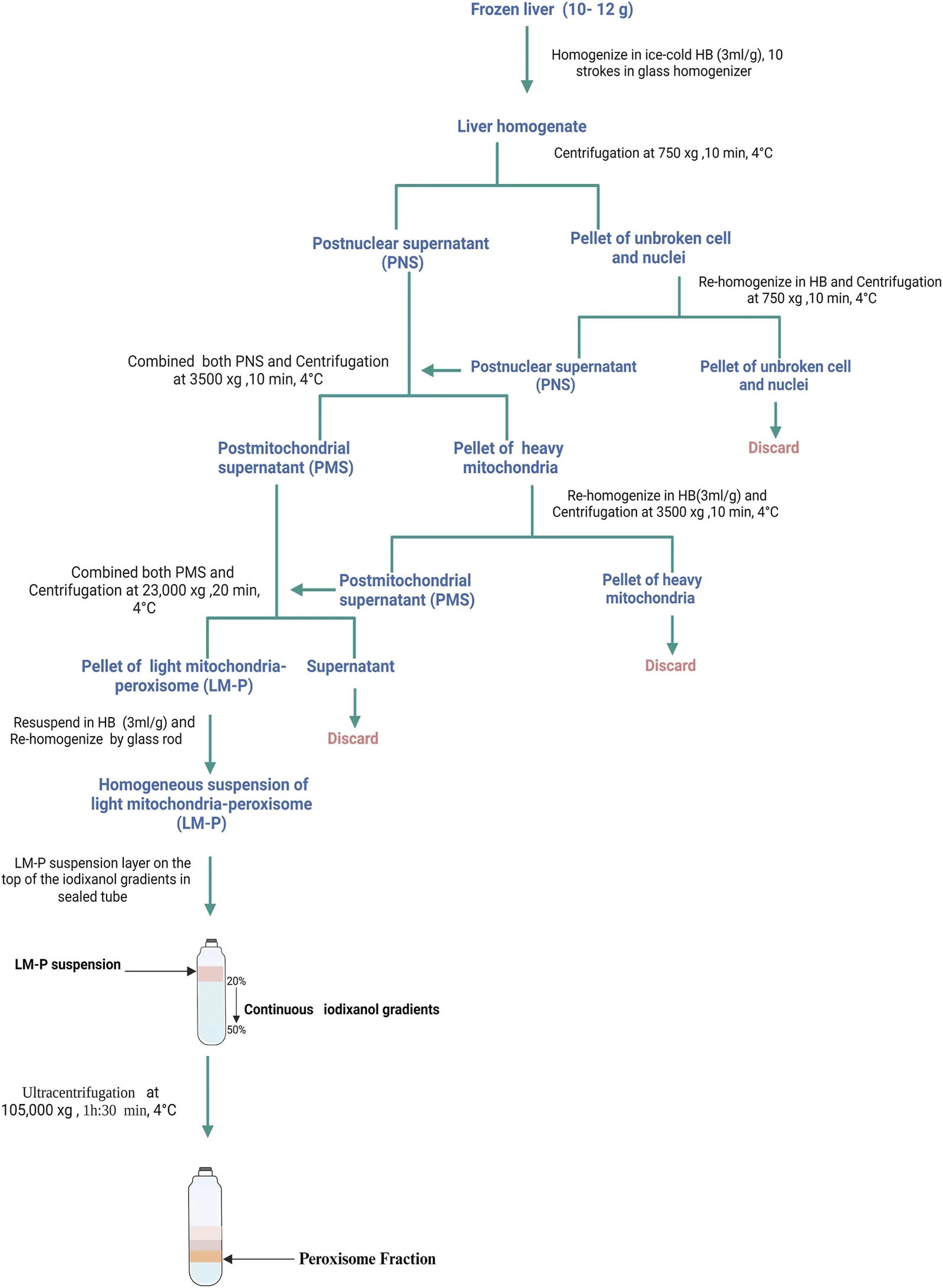
Scheme protocol for the isolation of peroxisomes from frozen liver in high-fat diet (HFD)-fed rats.

#### 2.2.11 Fatty acid extraction from serum

FA was extracted according to the Lewis method (Cavonius et al., 2014), (Lewis et al., 2000). The internal standard (ISTD) was prepared by dissolving 5 mg of methyl nonadecanoate in 10 mL of n-hexane. A volume of 400 μL serum was transferred to a 15-mL test tube and mixed with 2 mL of the solvent methanol:chloroform: hydrochloric acid (10:1:1). Then, the mixture was vortexed for 1 min and incubated for 2:30 h at 85 °C and 700 rpm in a water bath shaker. After cooling to room temperature, FA was extracted by adding 2.0 mL hexane–chloroform (4:1) and 1 mL, vortexed for 1 min, and centrifuged at 20 °C and 3000 rpm for 5 min. The clear supernatant was withdrawn using a Pasteur pipette and transferred to 4 mLamber. The n-hexane/chloroform was evaporated with a gentle stream of nitrogen at room temperature, repeating the extraction a total of three times. Finally, the extract was reconstituted in 0.5 ml of n-hexane and a volume of 170 µL of extract and 30 µl of internal standard was placed in vail and injected into Gas chromatography/mass spectrometry (GC-MS) for analysis (Figure 4).

**FIGURE 4:**
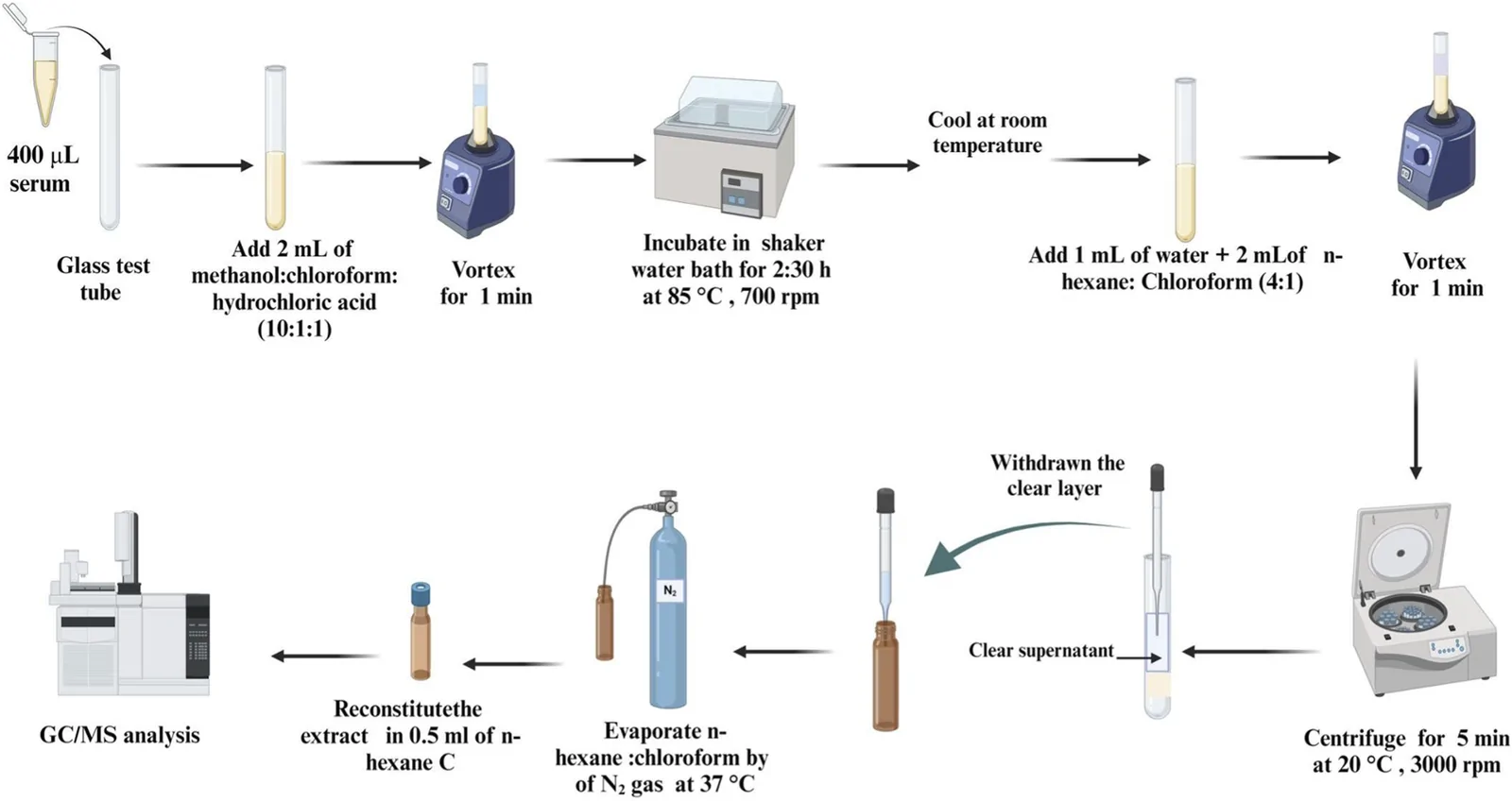
Fatty acid extraction from serum in high-fat diet (HFD)-fed rats.

### 2.3 Statistical analysis

The data were analyzed using GraphPad Prism 8 (GraphPad Inc., La Jolla, CA, United States of America). Data are shown as the means ± standard errors of the means (means ± SEM). A one-way ANOVA was used to assess the significant differences between different groups followed by Tukey’s *post hoc* test. Differences with *p* < 0.05 were considered statistically significant.

## 3 Results

### 3.1 Effect of carotenoids on body weight and abdominal fat

As shown in Figure 5, the rats fed HFD during the obesity induction period (12 weeks) showed a significant increase in body weight (p < 0.01) compared to the rats fed a normal diet (ND). In addition, the obese rats treated with carotenoids ether by Zi or Ly had no significant difference observed in body weight compared to the HFD group (Figure 5A). Abdominal fat was also significantly (p < 0.01) increased in the HFD group compared to the ND group, while the HFD + Zi and HFD + Ly had less abdominal fat compared to the HFD group, but not statistically significant (Figure 5B).

**FIGURE 5:**
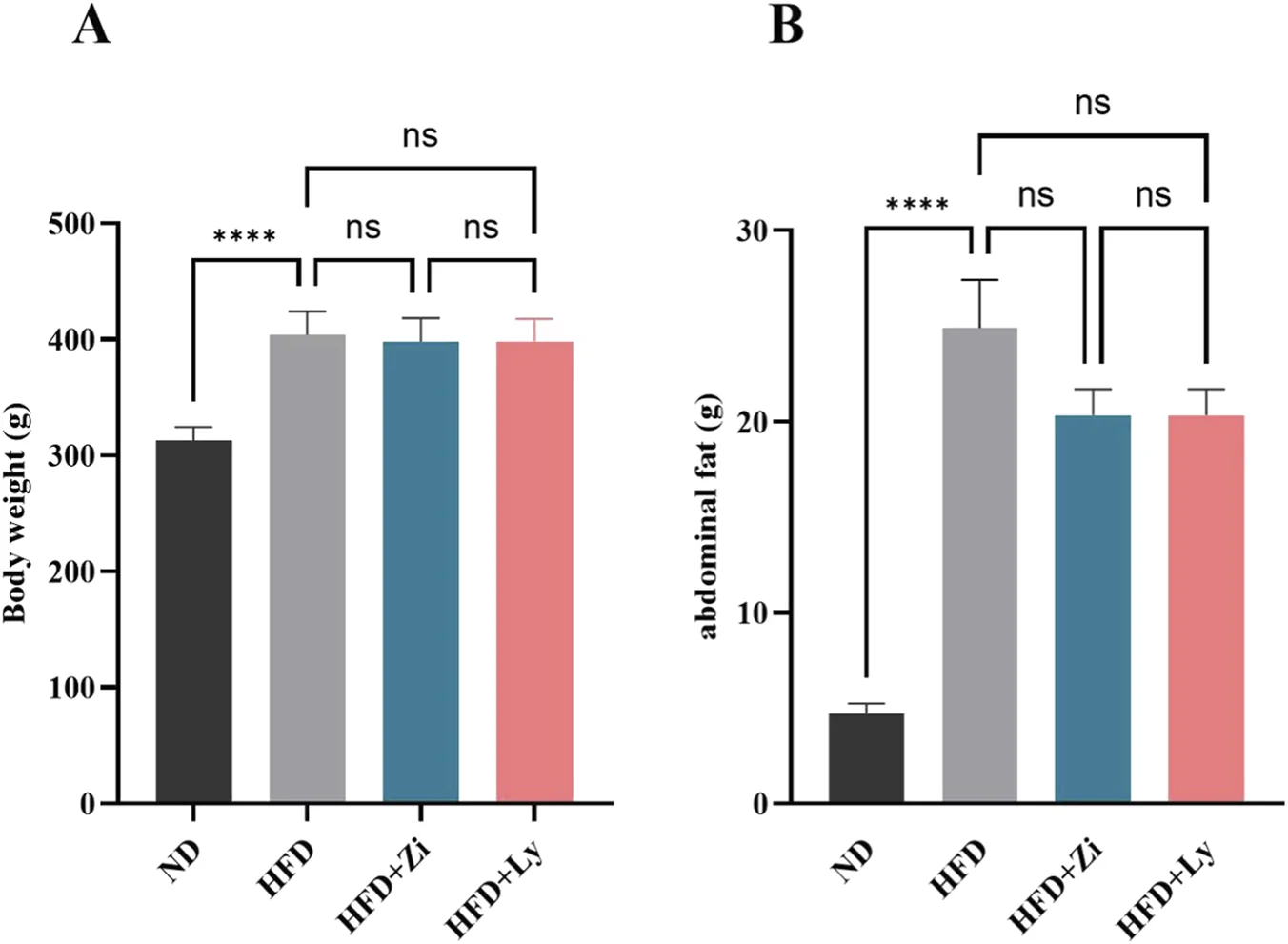
Effects of carotenoids (Zi and Ly) on body weights **(A)** and abdominal fat **(B)** in high-fat diet (HFD)-fed rats. Values are expressed as mean ± SEM (n = 10) (*p < 0.05; **p < 0.01; ***p < 0.001).

### 3.2 Effect of carotenoids on redox homeostasis biomarkers

Hepatic CAT activity had a significantly lower level in the HFD group compared to the ND group (p < 0.001). In addition, CAT levels were significantly increased in the groups treated with carotenoids (Zi, Ly) compared to the HFD group (p < 0.001) (Figure 6A). Moreover, SOD levels were lower in the HFD group compared with ND group, although there was no significant difference. Interestingly, the groups receiving Zi and Ly showed a significant increase in SOD levels (p < 0.001) compared with the HFD group (Figure 6B). On the other hand, the levels of MDA were significantly higher in HFD group compared with the ND group (p < 0.001), while they were significantly decreased in the Zi and Ly treated rats compared with the HFD group (p < 0.001) In addition, no significant difference was observed between HFD + Zi and HFD + Ly in the levels of CAT, SOD and MDA (Figure 6C).

**FIGURE 6:**
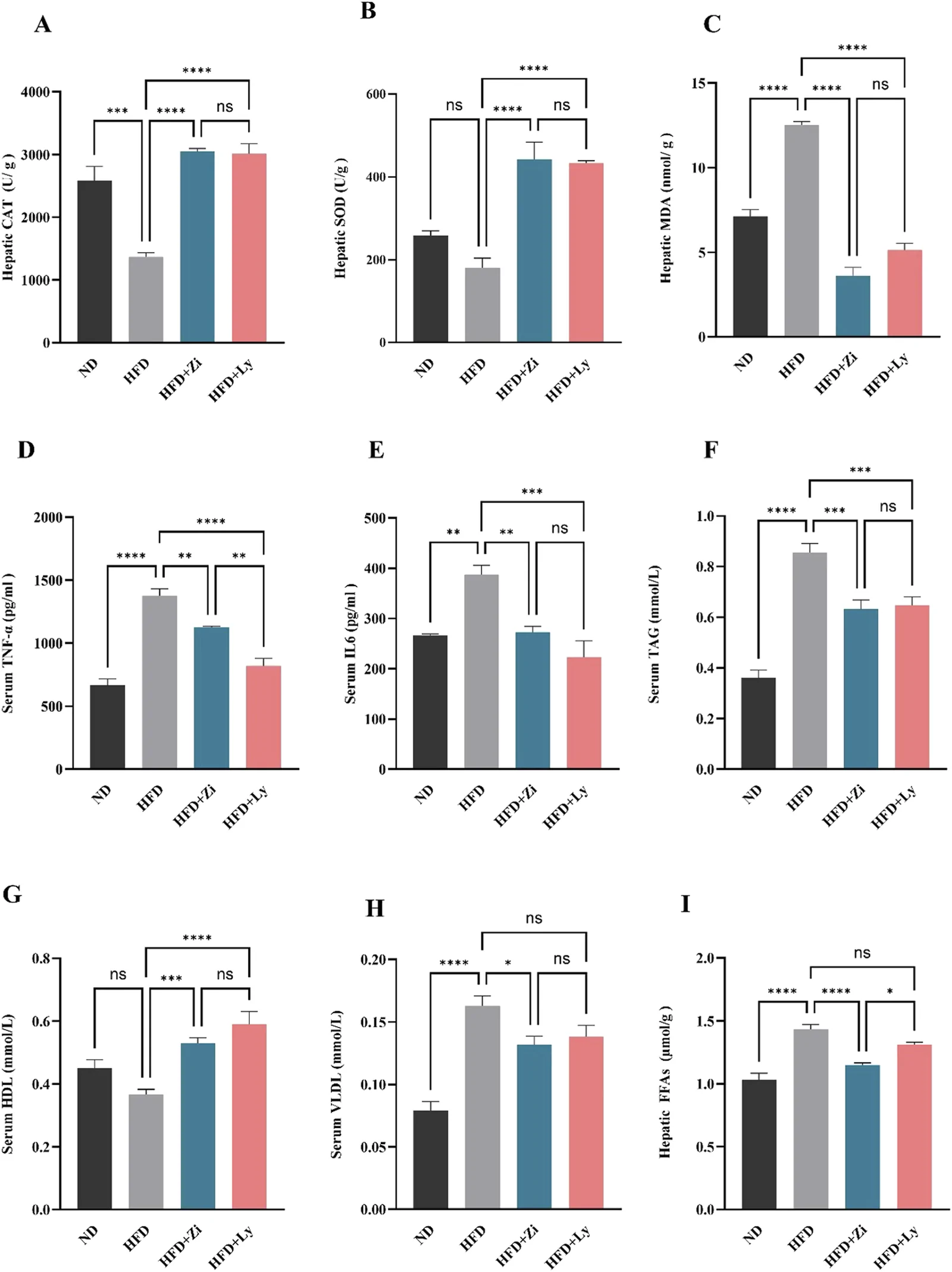
Effect of carotenoids (Zi and Ly) on redox homeostasis biomarkers, serum lipids and hepatic Free fatty acid (FFAs) in high-fat diet (HFD)-fed rats. **(A)** Catalase (CAT); **(B)** superoxide dismutase (SOD); **(C)** malondialdehyde (MDA); **(D)** tumor necrosis factor-α (TNF- α); **(E)** interleukin-6 (IL -6); **(F)** triacylglycerol (TAG); **(G)** High-density lipoprotein (HDL); **(H)** very low-density lipoprotein (VLDL) and **(I)** free fatty acids (FFA). Values are expressed as mean ± SEM (n = 5) (*p < 0.05; **p < 0.01; ***p < 0.001).

### 3.3 Effect of carotenoids on proinflammatory cytokines

Serum levels of TNF-α and IL-6 were significantly increased in the HFD group compared with the ND group (p < 0.01). However, TNF-α levels were significantly decreased after Zi and Ly administration compared with HFD group (Figure 6D). In addition, the groups treated with Ly had significantly lower TNF-α levels compared to the Zi treated groups. Moreover, IL-6 levels were significantly decreased in both the HFD + Zi and HFD + Ly groups compared with HFD group, while IL-6 levels were lower in the HFD + Ly group compared to HFD + Zi but there was no statistical difference, (Figure 6E).

### 3.4 Effect of carotenoids on serum lipids

The effects of Zi and Ly on serum TGA, HDL and VLDL levels in HFD-fed rats are shown in Figure 6. TGA levels in the HFD group were significantly higher than in the ND group (p < 0.001). In contrast, the treated groups (HFD + Zi and HFD + Ly) had significantly lower TAG levels compared with the HFD group (Figure 6F). In addition, HDL levels were reduced in the HFD group compared to the ND group with no significant differences. In addition, HDL levels showed significant increases in the treated groups (HFD + Zi and HFD + Ly) compared with the HFD group (Figure 6G). On the other hand, VLDL levels were found to be significantly higher in the HFD group than in the ND group (p < 0.001). In addition, the HFD + Zi and HFD + Ly groups had low VLDL levels compared with the HFD group, especially HFD + Zi, which were significantly decreased (p < 0.05) (Figure 6H).

### 3.5 Effect of carotenoids on hepatic free fatty acid (FFAs)

As illustrated in Figure 6, the levels of hepatic FFAs were significantly increased in the HFD group compared with the ND group (p < 0.001). However, after the intervention with Zi and Ly, FFA levels decreased in both groups compared to the HFD group. It is noteworthy that the FFA levels increased significantly in the HFD + Ly group compared with the HFD + Zi group (Figure 6I).

### 3.6 Effect of carotenoids on peroxisome function

As illustrated in Figure 7, peroxisomal catalase levels were decreased in the HFD group compared with the ND group, with no significant differences. Furthermore, after administration of Zi and Ly, peroxisomal catalase levels were significantly increased in both groups compared with the HFD group (p < 0.05) (Figure 7A). Additionally, Acyl CoA oxidase levels were significantly decreased (p < 0.001) in the HFD group compared with ND group. However, after intervention with Zi and Ly, the level of Acyl CoA oxidase was increased compared to the HFD group, particularly, HFD + Zi and which was significantly increased compared to the HFD group (p < 0.05) (Figure 7B). Notably, there was no significant difference between HFD + Zi and HFd + Ly in peroxisomal catalase Acyl CoA oxidase levels. Moreover, the FAOS activity was significantly decreased (p < 0.001) in the HFD group compared with the ND group. In contrast, the FAOS activity levels were increased in HFD + Zi while HFD + Ly has no significant difference compared with the HFD group (Figure 7C). In addition, the group that administered Zi had a higher level of FAOS compared with Ly treated group.

**FIGURE 7:**
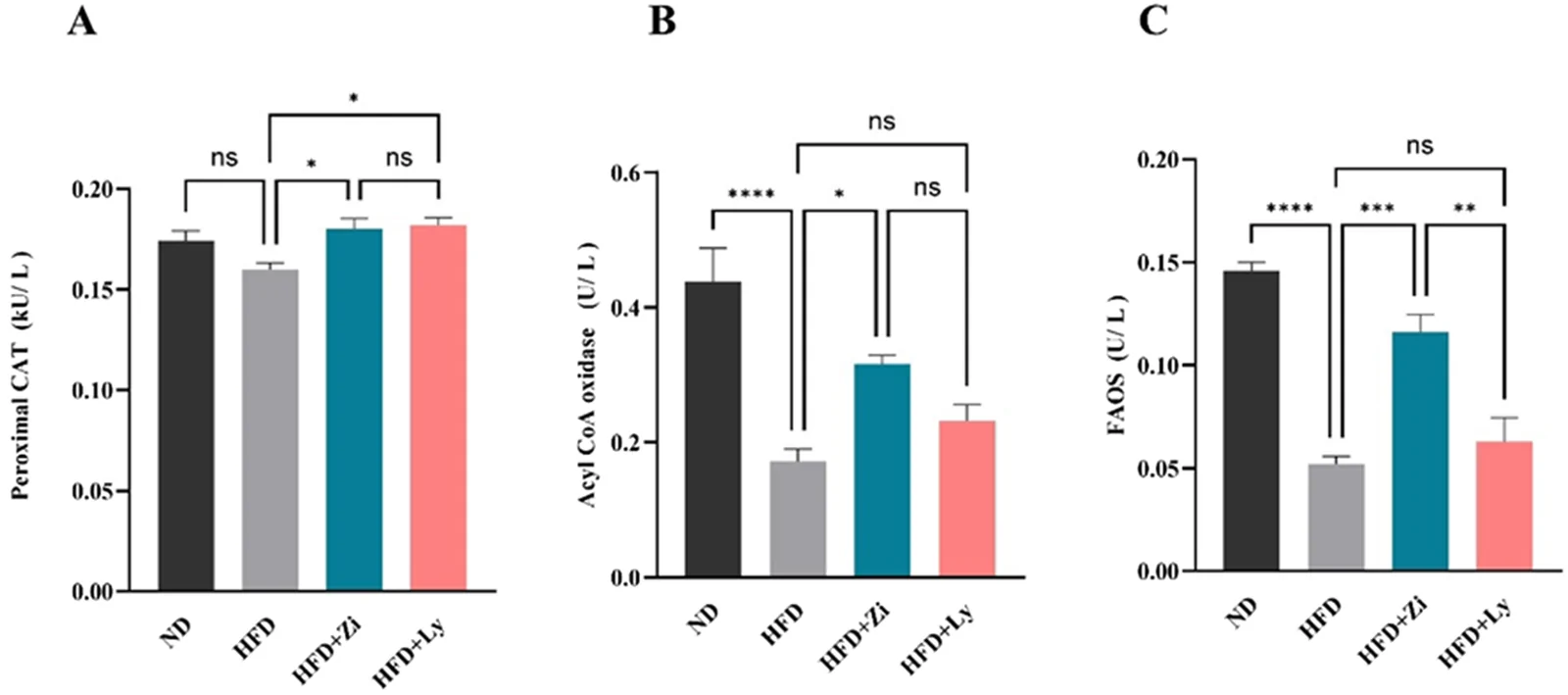
Effect of carotenoids (Zi and Ly) on peroxisome function **(A)** peroxisomal Catalase; **(B)** acyl CoA Oxidase; **(C)** fatty acid oxidation system (FAOS). Values are expressed as mean ± SEM (n = 5) (*p < 0.05; **p < 0.01; ***p < 0.001).

### 3.7 Fatty acid profile in serum

FFA profiles were analyzed by GC-MS. As shown in Figure 8, the chromatogram of the ND group represented five types of FFAs that were detected, including two saturated fatty acids palmitic acid (C16:0) and stearic acid (18:0), and three unsaturated fatty acids; one monounsaturated fatty acid, oleic acid (C18:1), and two polyunsaturated fatty acids; linoleic acid (C18:2) and arachidonic acid (C20:4), as shown in Figure 8A.It is worth noting that some peaks were not shown in the chromatogram while the areas integration were registered by the data system, due to the low concentration of the samples. On the other hand, thirteen different FFAs were identified in the chromatogram of the HFD group, including five saturated fatty acids; myristic acid (C14:0), pentadecanoic acid (C15:0), palmitic acid (C16:0), heptadecanoic acid (C17:0), and stearic acid (18:0), and eight unsaturated fatty acids; two monounsaturated fatty acids palmitoleic acid (C16:1) and oleic acid (C18:1) and six polyunsaturated fatty acids linoleic acid (C18:2), eicosatrienoic acid (C20:3), arachidonic acid (C20:4), eicosapentaenoic acid (C20:5), docosapentaenoic acid (C22:5), and docosahexaenoic acid (C22:6) (Figure 8B). The chromatograms in Figures 8C,D show the fatty acids that were detected in the treated groups (HFD + Zi, HFD + Ly) respectively. There were thirteen FFAs detected, similar to the HFD group but differed in the quantities and percentages of FFAs.

**FIGURE 8:**
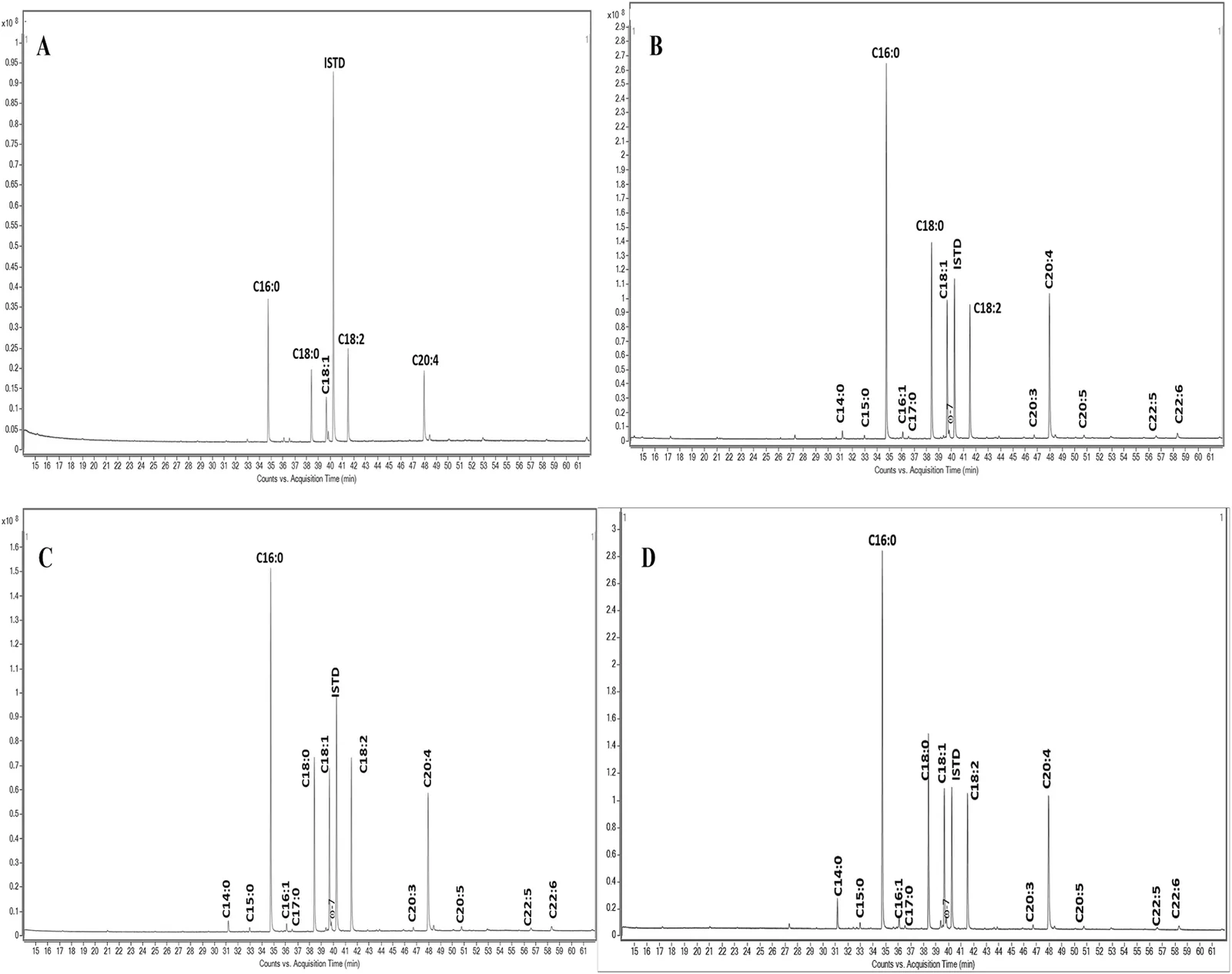
GC-MS chromatogram of fatty acids in the serum of high-fat diet (HFD)-fed rats. **(A)** Noraml diet (ND) group **(B)** HFD group **(C)**, HFD + Zi group and **(D)** HFD + Ly group. Myristic acid (C14:0), pentadecanoic acid (C15:0) palmitic acid (C16:0), heptadecanoic acid (C17:0), stearic acid (C18:0), palmitoleic acid (C16:1), oleic acid (C18:1), linoleic acid (C18:2), eicosatrienoic acid (C20:3), arachidonic acid (C20:4), eicosapentaenoic acid (C20:5), Docosapentaenoic acid (C22:5) and Docosahexaenoic acid (C22:6).

The percentages of fatty acids composition of all groups are given in Table 2. In the ND group, the predominant fatty acids were unsaturated fatty acids (66.10%), followed by saturated fatty acids (33.90%). In contrast, the percentage of saturated fatty acids in the HFD group was higher than in the control group (39.18%), and the percentage of unsaturated fatty acids was lower (60.82%) than in the ND group. However, after the administration of Zi the percentage of saturated fatty acid was decreased in HFD + Zi (35.88%) compared with HFD group while the HFD + Ly group had little increase (40.9%). In addition, the percentage of unsaturated fatty acids in HFD + Zi was higher than HFD group (64.12%) and lower in HFD + Ly (59.01%). Finally, it is important to mention that in all groups, the common saturated fatty acids were palmitic acid (C16:0) and stearic acid (18:0) while the predominant unsaturated fatty acids were the polyunsaturated linoleic acid (C18:2) and arachidonic acid (C20:4). Moreover, the ratio of unsaturated to saturated fatty acids (ΣUFA/ΣSFA) was higher in the ND group (1.95) than the HFD group (1.55). In contrast, the ratio (ΣUFA/ΣSFA) was higher in HFD + Zi (1.79) and lower in HFD + Ly (1.44) compared with HFD group.

**TABLE 2:** Fatty acid composition in serum of high-fat diet (HFD)-fed rats.

Fatty acid	Group			
	ND	HFD	HFD + Zi	HFD + Ly
% Saturated fatty acids (SFAs)				
Myristic acid (C14:0)	0.36	0.78	1.07	1.17
Pentadecanoic acid (C15:0)	1.04	0.55	0.67	0.62
Palmitic acid (C16:0)	18.08	21.38	20.34	21.56
Heptadecanoic acid (C17:0)	1.24	0.32	0.37	0.44
Stearic acid (C18:0)	13.17	16.11	13.43	14.59
% Unsaturated fatty acids (SFAs)				
Palmitoleic acid (C16:1)	1.23	1.03	0.56	1.83
Oleic acid (C18:1)	7.91	11.16	11.85	10.55
Linoleic acid (C18:2)	32.17	21.48	26.36	21.91
Eicosatrienoic acid (C20:3)	0.25	0.66	0.72	0.82
Arachidonic acid (C20:4)	23.20	22.35	20.84	20.91
Eicosapentaenoic acid (C20:5)	0.00	0.79	0.94	0.71
Docosapentaenoic acid (C22:5)	0.08	1.20	1.16	1.03
Docosahexaenoic acid, (C22:6)	1.26	2.11	1.69	1.68
ΣSFA	33.90	39.18	35.88	40.99
ΣUSFA	66.10	60.82	64.12	59.01
ΣUSFA/ΣSFA	1.95	1.55	1.79	1.44

The fatty acid composition was expressed as a percentage of the total identified fatty acids; % FAs was calculated by dividing individual FA concentration by the total concentration of fatty acids and multiplying by 100; ΣSFA = total saturated fatty acids; ΣUFA = total unsaturated fatty acids.

## 4 Discussion

Obesity is a complex disease defined as excess body fat and is a significant health problem worldwide. It is a critical factor in developing metabolic abnormalities in lipid metabolism, oxidative stress and chronic inflammation (Klop et al., 2013), (Leung et al., 2015), (Ligibel et al., 2015), (Scherer and Hill, 2016), (Varra et al., 2024a). During obesity, uncontrolled adipose tissue enlargement is associated with impaired fat metabolism, including excessive lipolysis, which leads to increased production and secretion of free fatty acids (FFAs) into the bloodstream (Sears and Perry, 2015). Peroxisomes are active organelles that play a key role in reactive oxygen species and lipid metabolism in cooperation with other metabolic organelles (Sugiura et al., 2017), (Wanders et al., 2018). Recent studies suggest that peroxisomes are also important for regulating energy homeostasis and that disruption of peroxisomal functions increases the risk of obesity and its complications (Kleiboeker and Lodhi, 2022b).

This study employed a technique to isolate peroxisomes and examined their activity, function, and susceptibility to a high-calorie diet, as well as the role of natural compounds—by virtue of their chemical properties—in enhancing their performance. Over the last 10 years, investigations have revealed that organelle dysfunction is a key factor in several diseases, and peroxisomes are increasingly recognized as vital components in both normal physiology and pathological states; however, techniques for isolating them for study remain limited. Until our recent publication (Baz et al., 2023) all techniques for isolating peroxisomes depended on the use of fresh tissue, with the exception of Alvarez et al. (1992), who successfully isolated peroxisomes from frozen human liver biopsies (Alvarez et al., 1992); no similar research has been conducted since, despite the successful isolation of other organelles (nuclei, mitochondria) from frozen samples. Isolating fresh tissue requires considerable time and resources, and the procedure can compromise the biological integrity and metabolic activity of peroxisomes, making preservation of the whole frozen organ more advantageous. In our prior study (Baz et al., 2023), we addressed this gap by providing the first thorough, reproducible method for isolating peroxisomes from frozen rat liver, supported by catalase enrichment, purification fold, and yield relative to mitochondrial, microsomal, and lysosomal benchmarks. In this investigation, we employed the previously validated technique unchanged and confirmed peroxisomal enrichment by quantifying catalase and ACOX activities in the purified fraction. The Methods section now explicitly cites Baz et al. (2023) and does not present the method as having been originally developed here.

Carotenoids such as Zi and Ly are considered one of the most abundant natural pigments (Wu et al., 2022). They offer various benefits for human health, such as antioxidant, anti-inflammatory, cardioprotective and neuroprotective properties (Khan et al., 2021c), (Lee et al., 2023). Zi (100 mg/kg/day) and Ly (25 mg/kg/day) doses were selected based on established rodent safety and efficacy profiles rather than weight-matching, reflecting their distinct chemical structures: zeaxanthin’s hydroxyl groups confer greater polarity and aqueous-phase antioxidant reactivity (Kumar et al., 2024b), whereas lycopene’s hydrocarbon structure favors lipophilic tissue accumulation, including crossing the blood–brain barrier (Guo et al., 2019). The Ly dose falls within the effective range used in prior rodent oxidative stress studies (Bedir et al., 2021), while zeaxanthin’s wider safety margin (no adverse effects up to 500–1000 mg/kg/day (Edwards, 2016)) supports its higher dose. Both carotenoids converge on shared anti-obesity pathways (PPARγ/C/EBPα/SREBP1-c downregulation, AMPK-mediated browning (Bilikozen Aygun et al., 2026)), but differ in relative potency: Zi more effectively enhanced antioxidant and peroxisomal activity, while Ly was more effective anti-inflammatorily—supporting distinct, only partially overlapping mechanisms rather than a dose-equivalent pathway.

HFD can induce obesity in animals and humans in agreement with the previous study, the induction of obesity in rats by feeding HFD was shown to be successful after 12 weeks (Blüher, 2019), (Syarif et al., 2024), (Baz et al., 2022), (Noreen et al., 2025a), (Xia et al., 2024) However, compared to the ND group, each rat fed HFD throughout the 12-week induction period had a significantly higher body weight (Gooda Sahib Jambocus et al., 2016). On the other hand, as shown in Figure 5, we observed no significant change in body weight and fat mass and a slight decrease in body weight and abdominal fat mass during treatment with Zi and Ly, which was due to the continuous feeding of HFD during the treatment period.

Redox homeostasis is a vital and dynamic mechanism that controls several biological processes and events by maintaining the equilibrium between reducing and oxidizing reactions in cells (Le Gal et al., 2021). Oxidative stress is important in the pathophysiology of obesity (Jin et al., 2023). In addition, HFD-induced obesity has been shown to lead to lipid peroxidation, which in turn causes oxidative damage by generating MDA, reducing the activity of antioxidant enzymes such as SOD and CAT, and depleting endogenous antioxidants (Agil et al., 2016), (Annamalai et al., 2017), (El Ayed et al., 2017). Peroxisome is one of the most oxidized cellular organelles and a major source of reactive oxygen species (ROS) generation in cells (Smirnoff and Arnaud, 2019). Peroxisomes have a sophisticated antioxidant system that maintains the balance of ROS and allows them to tightly control organelle functions, metabolism and signalling pathways (Sandalio et al., 2021). In the current study, rats fed an HFD exhibited a significant decrease in CAT and SOD levels with a parallel increase in MDA, which is in agreement with prior findings (Lasker et al., 2019). After administration of Zi and Ly, the levels of CAT and SOD were significantly increased, while the MDA levels were significantly decreased compared with the HFD group. It is commonly approved that carotenoids act as electron transporters and are essential for the protection of tissues, organs and cells from oxidative damage caused by lipid peroxide free radicals (Milani et al., 2017). These compounds exert their therapeutic effects by eliminating superoxide radicals and modulating the expression of inflammatory markers such as NF-κB and ICAM-1, thereby reducing the biochemical imbalances associated with HFD (Tuzcu et al., 2017), (Eze et al., 2018). This decrease in MDA levels, along with the enhancement of intrinsic enzymatic function, indicates that these antioxidants proficiently halt lipid peroxidation chain processes and maintain the cellular redox state (Abd El-Gawad et al., 2019), (Noreen et al., 2025b). According to the findings of previous studies (Bouyahya et al., 2021), (Tuzcu et al., 2017), (Albrahim and Alonazi, 2021), Zi and Ly can exert antioxidant properties by directly suppressing reactive oxygen species. In one study, Zi supplementation was found to modulate oxidative stress in rats fed a HFD (Tuzcu et al., 2017). In addition, administration of Ly in diethylnitrosamine-induced hepatocarcinogenesis in Wistar rats decreased MDA levels while increasing SOD and CAT activity (Sahin et al., 2014). Ly has antioxidant properties that stimulate the transcription of genes containing antioxidant response elements, thereby strengthening cellular defense systems (Lee et al., 2019). Moreover, studies demonstrate that the hydroxyl radical scavenging capability of Zi surpasses that of other carotenoids, providing a potent means of counteracting cellular oxidative damage (Mitra and Bera, 2019). The observed enhancement of peroxisomal β-oxidation with Zi and Ly supplementation may be partially explained by two literature-supported mechanisms, although direct molecular assays were not performed in this study. Peroxisomal β-oxidation generates hydrogen peroxide (H_2_O_2_) as a byproduct of acyl-CoA oxidase activity, and catalase serves as the organelle’s primary local defense against this oxidative byproduct. Reduced catalase activity has been shown to cause peroxisomal ROS accumulation and membrane leakage, compromising organelle stability. Zi and Ly support antioxidant defenses both directly, by scavenging free radicals, and indirectly, by upregulating catalase, SOD, and glutathione peroxidase via the Nrf2 pathway—Zi specifically promotes Nrf2 nuclear translocation and glutathione synthesis (Zou et al., 2014). Given that peroxisomal fatty acid metabolism continuously generates H_2_O_2_, this antioxidant activity may help preserve peroxisomal redox homeostasis and enzymatic integrity under HFD-induced oxidative stress. Ly has also been shown to upregulate hepatic PPARα and catalase/SOD activity (Shafe et al., 2024); since PPARα regulates key peroxisomal β-oxidation enzymes (Tahri-Joutey et al., 2021), this suggests a plausible transcriptional mechanism. We propose that the improvements in peroxisomal β-oxidation probably result from a combination of Nrf2-mediated antioxidant mechanisms specifically the protection of catalase activity against peroxisomally generated H_2_O_2_ and potential transcriptional regulation via PPARα, rather than a primary effect on peroxisomal biogenesis, which was not examined here (e.g., via PEX gene expression or peroxisome number/morphology). This molecular hypothesis requires direct validation in future studies through PEX gene expression analysis, PPARα reporter assays, or Nrf2 pathway inhibition experiments.

Obesity was considered a low-grade systemic inflammatory condition (Sam et al., 2009). In obesity, adipocytes increase in number and size, especially in visceral adiposity, which is associated with infiltration of macrophages, which in turn leads to the production of proinflammatory cytokines such as TNF-α and IL -6 (Sam et al., 2009), (Jager et al., 2007), (Khanna et al., 2022), (Savulescu-Fiedler et al., 2024). Peroxisomes are vital metabolic organelles that play an essential role in the production and degradation of reactive species and complex lipids. They are important regulators of inflammation and immune responses (Di Cara et al., 2019). The present study showed that the high-fat diet could increase TNF-α and IL -6 in the HFD group compared to the ND group, which is consistent with previous studies (Albrahim and Alonazi, 2021), (Yazdani et al., 2022). However, after Zi administration, TNF-α and IL -6 decreased markedly compared to the HFD group, which is due to the anti-inflammatory effect of Zi demonstrated in many previous studies (Bian et al., 2012), (Nidhi et al., 2015), (Shim and Shim, 2019), (Zhou et al., 2018). Also, Ly supplementation reduces TNF-α and IL -6 as it has an anti-inflammatory effect, which effectively reverses the changes caused by HFD (Albrahim and Alonazi, 2021), (Pierine et al., 2014b). The elevation of TNF-α and IL-6 in HFD subjects indicates the inflammation characteristic of diet-induced obesity, which is associated with the peroxisomal dysfunction reported in this study. Diminished FAOS and catalase activity lead to fatty acid buildup and increased ROS/MDA, which subsequently promote cytokine synthesis (Gómez-Ambrosi, 2023), (Varra et al., 2024b).

Serum lipid profiles are useful indicators for the development of metabolic diseases associated with obesity (Che et al., 2019). Previous studies have shown that HFD is associated with dyslipidemia characterized by increased VLDL and TGA levels and decreased HDL (Mamun et al., 2019), (Sifat et al., 2020). The current study showed that rats fed HFD had high TGA, VLDL and low HDL levels, suggesting the development of dyslipidemia. Peroxisomal lipid metabolism is an essential signalling pathway associated with lifestyle-related diseases (Kozawa et al., 2011). A defect in this organelle leads to an accumulation of lipids inside the cell. On the other hand, HFD + Zi and HFD + Ly groups had observed an increase in HDL levels and a decrease in TGA and VLDL levels, which is in agreement with the previous studies (Wu et al., 2022; Khan et al., 2021c). According to our results, supplementing with Zi and Ly can help rats fed a HFD to avoid dyslipidemia and improve their lipid profile. In addition, Zi and Ly can activate various nuclear receptors that control the transcription of several signalling pathways involved in lipid metabolism (Sharoni et al., 2012).

About 60% of hepatic fat is derived from free fatty acids (FFAs), which are released into the blood by adipocytes (Donnelly et al., 2005) (Jung and Choi, 2014). In most organisms, fatty acids are mainly broken down via the β-oxidation cycle. Impaired fat oxidation in obesity leads to increased hepatic fat accumulation and an increased risk of metabolic syndrome (Muñoz et al., 2018). In the current study, rats fed HFD exhibited significantly increased levels of hepatic FFAs compared with the NDgroup, which is consistent with previous findings (Ligibel et al., 2015), (Elvira-Torales et al., 2019), (Wang et al., 2017). However, after the intervention with Zi and Ly, FFA levels were reduced, especially in the HFD + Zi group, which were significantly reduced compared to the HFD group, consistent with previous studies (Tuzcu et al., 2017), (Wang et al., 2020). This can be explained by the role of Zi and Ly as antioxidants. Oxidative stress resulting from obesity can lead to peroxisome dysfunction, which causes decreased fatty acid oxidation and hence, increases lipid accumulation and impaired liver function (Nassir et al., 2015). For this reason, the use of Zi and Ly as an antioxidant has been investigated as a potential mechanism for the prevention and treatment of fatty liver (Murillo and Fernandez, 2016).

Peroxisomes play an essential role in β-oxidation of very long chain fatty acids, the production and detoxification of H_2_O_2_ and the synthesis of ether phospholipids and bile acids (He et al., 2020b). The most common peroxisomal antioxidant enzyme is catalase, which efficiently eliminates H_2_O_2_ generated during peroxisomal β-oxidation to preserve cellular and peroxisomal redox balance (Walker et al., 2018). It is important to note that the function of catalase enzymes is not only to scavenge H_2_O_2_ produced inside the peroxisome but also to eliminate H_2_O_2_ coming from outside the cell. Peroxisomes thus act as H_2_O_2_ sinks or sources, which most likely depends on the cell type, its physiological state and the microenvironment (Lismont et al., 2019). In the present study, we isolated the peroxisomes and measured the enzyme catalase in the isolated fraction to investigate the function of the peroxisomes specifically and more precisely. Catalase is highly present in peroxisomes, so the presence of this enzyme is an indicator of peroxisome activity. In our study, the level of peroxisomal catalase was reduced in the HFD group compared with the ND group. After the administration of Zi and Ly, peroxisomal catalase levels were increased compared with the HFD group. An explanation for this is provided by the study by (Jiang et al., 2022), which suggests that obesity and HFD decrease peroxisomal function and biogenesis, which in turn increases liver damage including oxidative stress and inflammation. Since catalase is recognized as a critical element of the peroxisomal pathway for fatty acid oxidation, deficiency or inhibition of catalase activity may indicate that disruption of the peroxisomal β-oxidation process in this manner leads to significant disturbances in lipid metabolism (Crane and Masters, 1984).

The first step of peroxisomal β-oxidation is carried out by acyl-CoA oxidase, which catalyzes the desaturation of acyl-CoA to 2-trans-enoyl-CoA. Peroxisomal acyl-CoA oxidase (Acox) catalyzes the first and rate-determining step of peroxisomal β-oxidation of fatty acids and it is a major producer of hydrogen peroxide (He et al., 2020b), (Ferdinandusse et al., 2007b). Acox preferentially degrades straight-chain fatty acids involved in the oxidation of VLCFAs (Van Veldhoven, 2010). In this study, the level of this enzyme was measured in the isolated fraction, as it is one of the most important enzymes that triggers the β-oxidation reactions in the peroxisome. The measurement of this enzyme gives us an indication of the activity of the metabolic process in the peroxisome. Our results showed that the Acox level was significantly decreased in the HFD group compared with the ND group. In contrast, the Acox level was increased after the intervention with Zi or Ly compared to the HFD group. In addition, we found that Zi and Ly enhanced the level of Acox, but the mechanism remains unknown due to the limited studies and research concerned with peroxisomal Acox and its relationship to obesity, HFD and metabolic dysfunction. Therefore, it is imperative to conduct more research using other techniques to investigate peroxisomal β-oxidation pathways, such as cell signaling pathways.

The fatty acid oxidation system (FAOS) assay is an important method for measuring the overall activity of the peroxisomal β-oxidation spiral (Lazarow, 1981). In the present study, we found that FAOS activity was increased in HFD + Zi and HFD + Ly, especially in the HFD + Zi which was significantly increased. From our results, it appears that Zi and Ly affect the overall fatty acid oxidation system by increasing its activity, but the mechanisms, such as the Acox mentioned above, are still unknown.

Catalase, Acox, and FAOS were chosen as complementary functional markers of peroxisomal activity: catalase reflects antioxidant capacity by degrading β-oxidation-derived H_2_O_2_, while ACOX and FAOS reflect β-oxidation activity directly. Catalase also protects the β-oxidation system, including thiolase, from H_2_O_2_-induced inactivation. Additional markers of peroxisomal abundance, biogenesis, or purity (such as PMP70, PEX gene expression, and electron microscopy) were not assessed in this study and are recommended as vital pathways for future research to support a more comprehensive characterization of peroxisomal biogenesis and turnover. Interestingly, the results of catalase, peroxisomal Acox and FAOS suggest that HFD-induced obesity causes peroxisomal dysfunction, which is consistent with previous studies by (Piao et al., 2019b), who reported that peroxisome defects are associated with adipocyte dysfunction and impaired peroxisomal capability significantly increases oxidative stress and inflammation in adipocytes, which eventually makes obesity worse. Moreover, according to (Yao et al., 2023), targeting peroxisomal β-oxidation could be a promising way to improve hepatic steatosis and insulin resistance caused by obesity or diabetes. Peroxisomal β-oxidation may influence systemic metabolism and obesity by regulating lipid homeostasis in the liver and adipose tissue (Kleiboeker and Lodhi, 2022b). Importantly, Zi and Ly increased peroxisomal biogenesis and function via catalase, peroxisomal Acox and FAOS in the liver of rats fed an HFD, suggesting that the enhanced peroxisomal fitness induced by Zi and Ly may play an important role in protecting against metabolic complications of obesity.

In rodents, high-fat diets are commonly used to cause obesity, increase serum fatty acid levels and cause lipotoxicity in various organs (Liu et al., 2015). For example, FFA levels in the blood are associated with obesity, diabetes, cardiovascular disease and hepatic steatosis (Ichihara et al., 2020). The fatty acid concentration in tissues and serum can be identified and measured with high accuracy by gas chromatography (GC), which is a highly sensitive technique. The results showed that in comparison to the ND group, the HFD group had a higher percentage of saturated fatty acids. Moreover, the HFD group had a low percentage of unsaturated fatty acids compared to the ND group. This is consistent with a study by Kaikkonen, et al., which found a positive correlation between obesity, insulin resistance elevated blood pressure and saturated fatty acids with an even number of carbon atoms (Kaikkonen et al., 2021). While the HFD has a high content of saturated fatty acids, the predominant ones are palmitic acid (C16:0) and stearic acid (18:0). These fatty acids play an important role in lipotoxicity. Chen and coworkers (2016) reported that long-chain saturated fatty acids such as palmitic acid have a strong cytotoxic effect on insulin-producing cells, while short chain and unsaturated fatty acids are well tolerated (Chen et al., 2016). In addition, Piccolis et al. found that long chain unsaturated fatty acids such as oleic acid or arachidonic acid counteract the toxicity of palmitic acid. Additionally, the cellular lipotoxicity caused by saturated fatty acids leads to cell death and is considered one of the main causes of metabolic diseases (Piccolis et al., 2019). In contrast, our results showed that HFD + Zi had low percentages of saturated FFAs compared with the HFD group and high percentages of unsaturated FFAs compared with the HFD group. On the other hand, it was observed in the study that the group administrated Ly looked similar to the HFD group in percentage terms and did not show any noticeable effects compared to the HFD group. It is noteworthy that polyunsaturated linoleic acid (C18:2) and arachidonic acid (C20:4) were the predominant unsaturated fatty acids. Additionally, we observed that the administration of Zi can increase the content of unsaturated fatty acids, especially polyunsaturated fatty acids, and reduce the content of saturated fatty acids. Previous studies have shown that patients who had lost weight had significantly lower levels of selected saturated fatty acids and increased levels of polyunsaturated fatty acids (Wrzosek et al., 2022).

It is important to do the GC/MS test to investigate the accumulation of VLFAs in the serum, which is related to the peroxisomal disorder. VLCFAs with a carbon chain length ≥22 are only metabolized in peroxisomes by β-oxidation. Therefore, measuring VLCFAs in plasma/serum is an important second-line investigation in the diagnosis of peroxisomal disorders (Carragher and Champion, 2014). *In vivo*, free VLCFAs are typically highly toxic, and their oxidative metabolism relies on peroxisomal β-oxidation (Lu et al., 2022b). VLCFAs containing C22 and C24 are widely distributed in the body. Ultra long chain fatty acids (ULCFAs) are FAs with C ≥ 26 that are found in certain tissues such as the retina, skin, testis, meibomian gland and brain (Sassa and Kihara, 2014). Therefore, the presence of VLCFAs serum or tissue in high concentrations is considered harmful and an indicator of a major defect in the peroxisome. In our study, we found only very low percentage, almost negligible of VLCFAs. The β-oxidation 2, the only dedicated VLCFAs were docosapentaenoic acid (C22:5) docosahexaenoic acid (C22:6). The percentage of docosapentaenoic acid (C22:5) and docosahexaenoic acid (C22:6) varied between groups but did not exceed the highest percentage of 1.2% and 2.5% in all groups, respectively. It is very important to mention that high plasma and tissue VLCFA levels have been found only in peroxisomal disorders. This is a genetically heterogeneous group but in disorders of both peroxisomal biogenesis (such as Zellweger syndrome) and defects in individual peroxisomal proteins (the most common of which is X-linked adrenoleukodystrophy (X-ALD) (Carragher and Champion, 2014).

Based on our results, we rule out the occurrence of a major defect at the peroxisomal level, but a defect may occur in the β-oxidation of the VLCFAs process, which is supported by the results of catalase, Acyl CoA oxidase and FAOS. Therefore, we recommend conducting an analysis of VLCFAs in the liver to ensure that any accumulation of VLCFAs is due to a defect in peroxisomal β-oxidation.

Overall, our study found that HFD and lack of physical activity in experimental rats lead to obesity caused by the accumulation of excess fat in adipose tissue, resulting in increased lipolysis and circulating FFAs, which are transported via the blood to the organs of the body, especially the liver, which is the central organ for fatty acid metabolism. This increase in fatty acids is eliminated by the esterification of FFAs to TAG, which is then secreted into the bloodstream in the form of VLDL, leading to an increase in lipid profiles and causing hyperlipidemia. On the other hand, the excess FFAs are eliminated by β-oxidation in the mitochondria and peroxisomes, but the oxidation process does not continue for a long time due to the accumulation of FFAs that exceed the capacity of mitochondria and peroxisomes to oxidize, leading to impaired β-oxidation and dysfunction mitochondria and peroxisomes. As a result of the overload of β-oxidation, ROS such as MDA and H_2_O_2_ were produced by inducing lipid peroxidation in the absence of antioxidant enzymes such as catalase and SOD. This led to oxidative stress and an increase in inflammatory markers like TNF-α and IL-6. Since peroxisomes are essential for mitochondrial health, the absence of peroxisomes in hepatocytes leads to a concerted effort to preserve mitochondrial function, including increased mitochondrial biogenesis. In this study, the effects of HFD on peroxisome function were noted by a decrease in marker enzymes of peroxisomes such as catalase, and in acyl CoA oxidase, which is the rate-limiting enzyme in a very long chain fatty acid β-oxidation and an overall decrease in the activity of fatty acid oxidation system (FAOS). In addition, supplementation with Zi and Ly affected the overall peroxisomal β-oxidation by increasing the marker enzymes catalase and acyl-CoA oxidase. Moreover, Zi and Ly were found to improve the complications that arise from obesity, including ROS, and inflammation and improve the oxidation process in the peroxisomes through the Zi and Ly antioxidant, anti-inflammatory. The primary limitation of the current work is the need for further investigation into how carotenoids and other natural agents affect lipid metabolism. Future projects should emphasize on optimizing the isolation of peroxisome from complex tissues such as adipose tissue and brain, and on applying VLCFA analysis, genetic techniques, and peroxisomal staining to develop a more comprehensive understanding of peroxisome functionality in different tissue types.

## 5 Conclusion

In this study, we found that HFD consumption was associated with weight gain, fat accumulation, dyslipidemia, and disturbances in the assessed inflammatory and oxidative stress parameters. Treatment with Zi and Ly was associated with lower levels of the inflammatory biomarkers TNF-α and IL-6, as well as reduced MDA levels, suggesting an improvement in lipid peroxidation status. Moreover, this study demonstrates for the first time that HFD-induced obesity critically impairs peroxisomal function, as evidenced by disrupted fatty acid β-oxidation and reduced peroxisomal catalase activity, along with imbalanced LCFAs and VLCFAs levels. Supplementation with Zi and Ly was associated with improvements in these parameters, including increased peroxisomal catalase activity, enhanced fatty acid β-oxidation, normalization of LCFA and VLCFA levels, and reductions in oxidative stress and inflammation. These changes may contribute to mitigating some of the metabolic disturbances associated with obesity. In addition, the results of the GC/MS analysis of serum fatty acids demonstrated that feeding HFD led to an increase in saturated fatty acids in serum relative to unsaturated fatty acids. Overall, the present study suggests that treatment of obese rats with both carotenoids could protect the body from the harmful effects of a HFD through antioxidant and anti-inflammatory properties. To our knowledge, there are no previous research studies on the effect of Zi and Ly on peroxisomal catalase function and obesity, which prompts us to conduct further research in the future.

## Data Availability

The raw data supporting the conclusions of this article will be made available by the authors, without undue reservation.
